# Correlation of chest CT severity score with clinical parameters in COVID-19 pulmonary disease in a tertiary care hospital in Delhi during the pandemic period

**DOI:** 10.1186/s43055-022-00832-x

**Published:** 2022-07-28

**Authors:** Swati Sharma, Anjali Aggarwal, Rajat K. Sharma, Elisheba Patras, Annu Singhal

**Affiliations:** 1 Department of Radiodiagnosis, Atal Bihari Vajpayee Institute of Medical Sciences and Dr. RML Hospital, New Delhi, 110001 India; 2 Department of Radiodiagnosis, St. Stephens Hospital, Doctor’s Hostel, Tis Hazari, New Delhi, 110054 India; 3 Department of Radiodiagnosis, St.Stephens Hospital, Tis Hazari, New Delhi, 110054 India

**Keywords:** COVID-19, CT severity score, Pandemic, Clinical

## Abstract

**Background:**

Since November 2019, the rapid outbreak of coronavirus disease 2019 (COVID-19) has become a public health emergency of international concern. COVID-19 disease is caused by a new variant of coronavirus, named as ‘severe acute respiratory syndrome coronavirus 2.’ Chest CT has a potential role in the diagnosis, detection of complications and in predicting clinical recovery of patients or progression of coronavirus disease 2019. Degree and severity of lung involvement can be assessed by 25 point CT severity score. This quantification plays an important role to modify the treatment plan at times in critically ill patient of COVID-19. Hence, the purpose of present study was to describe and quantify the severity of COVID-19 infection on chest computed tomography (CT) by 25-point CT severity score and to determine the relationship of CT severity score with clinical and laboratory parameters.

**Results:**

A total of 150 patients with COVID-19 disease were assessed. Mean age of the study group was 54.46 years (62.7% males and 37.3% females). The most common comorbidity present in the study group was diabetes mellitus, which was present in 17.3% cases. Severity of disease was significantly associated with age of the patient. CT severity score was positively correlated with lymphopenia and raised CRP, D-dimer and serum ferritin levels. A significant statistical correlation was found between CT severity grade and patient survival.

**Conclusions:**

This is a large comprehensive study, collecting data from 150 cases of COVID-19 pneumonia patients, in a tertiary care hospital in India to describe the correlation of CT severity score with clinical land laboratory parameters. Chest CT severity score correlates well with laboratory parameters and can aid in predicting COVID-19 disease outcome.

## Background

In December 2019, a novel Coronavirus (2019-nCoV) is reported to have surfaced from Wuhan in the Hubei region of China. This new variant of coronavirus was named ‘severe acute respiratory syndrome coronavirus 2 (SARS-CoV-2),’ and the disease it causes is referred to as coronavirus disease 2019 (COVID-19) [1, 2].

Coronavirus disease 2019 (COVID-19) is the first epidemic of the twenty-first century, almost 10 years after MERS outbreak in 2012 [1], and has become a pandemic [3].

COVID-19 disease is a highly infectious disease and spreads through respiratory droplets, contact, and the fecal–oral route. The incubation period of virus is approximately 2–14 days. Infection with SARS-CoV-2 causes respiratory illness in the form of severe pneumonia, intermittent fever, and cough. Symptoms of pharyngitis, rhinorrhea, and sneezing have been less commonly seen.

Currently, reverse transcription polymerase chain reaction (RT-PCR) is considered the gold standard for COVID-19 detection, but is limited by the time required to transport and prepare samples for testing, resulting in significantly delayed diagnostic times [4].

Chest computed tomography (Chest CT) is a rapid and easily available test that may aid in the diagnosis of COVID-19, especially in the current climate of overrun laboratories [4].

As recently reported in few studies, chest CT demonstrates typical radiologic features in majority of patients with COVID-19 disease, including bilateral GGOs in the lower lobes with a peripheral or posterior distribution, which further develops into the crazy-paving pattern and subsequent consolidation [5, 6]. Also, severity and prognosis of the disease can be assessed by imaging findings which supports clinicians in timely management.

At present, there are little data available correlating CT imaging features with systemic inflammatory markers in COVID-19 pneumonia patients.

Herein, the purpose of this study is to evaluate severity of COVID-19 pneumonia by quantifying CT severity score in confirmed cases of COVID-19 disease and to correlate CT severity score with clinical and laboratory parameters.

Although many of the previous studies have already described the chest CT imaging features of COVID-19 pneumonia, there is still a lack of large-sample CT imaging studies. This is a large comprehensive study, collecting data from 150 cases of COVID-19 pneumonia patients, in a tertiary care hospital in India to describe the correlation of CT severity score with clinical land laboratory parameters.

## Methods

### Study design

The present retrospective analysis was done on COVID-19 laboratory-confirmed cases admitted in St. Stephens Hospital, Tis Hazari, Delhi, for a period of 12 months from October 1, 2020, to October 1, 2021. To ensure the quality and integrity of clinical, and imaging data, here we included 150 patients with COVID-19 who had been admitted to our institution. The diagnosis of COVID-19 was made based on the World Health Organization interim guidance, wherein confirmed cases denoted were patients whose RT-PCR assay findings for nasal and pharyngeal swab specimens were positive [7].

The privacy and confidentiality of patients were observed as per norms. Informed consent was waived off as per ethics committee. Our institutional review board approved this descriptive study.

### Patient selection

#### Inclusion criteria

- All clinically suspicious patients of COVID-19 infection who are referred for chest CT evaluation and eventually diagnosed by means of available laboratory test.
- All laboratory-confirmed patients of COVID-19 infection who are referred for chest CT evaluation.

#### Exclusion criteria

- Patients with negative RT-PCR for SARS-CoV-2.
- Patients who are pregnant.
- Patients with lung surgery and lung tumor history
- Patients with any other causes of common bacterial or viral pneumonia.

### Data collection

We retrospectively collected the clinical and chest imaging data of COVID-19 pneumonitis cases. This included epidemiological data and clinical data including age, gender, laboratory data, comorbidities of patients, CT chest characteristics, CT severity score. After collection of all required data and careful medical chart review, the clinical and laboratory data of COVID-19 patients were compiled and tabulated.

### Analysis of laboratory findings

The following laboratory abnormalities on blood tests on admission were considered and correlated with CT severity score: C-reactive protein (CRP) (< 0.1 mg/dL), erythrocyte sedimentation rate (ESR) (0–10 mm per hour), white blood cell count (WBC) (4–10 × 10^3^/µL), D-dimer (0–200 ng/mL), serum ferritin (23.9–336 ng/mL), and lymphocyte count (1–3 × 10^3^/µL).

### CT assessment and data analysis

The patients were subjected for chest CT scan in axial view followed by multiplanar reconstruction in the coronal and sagittal planes. The scans were performed using a Philips incisive 128 MDCT scanner. All images were obtained with patients in the supine position. All images were reconstructed with a slice thickness of 0.625–1.250 mm with the same increment. The main scanning parameters were as follows:

**Table:** 

Tube voltage	120kvp
Automatic tube current modulation (tube current)	140–200mAs
Pitch	0.99–1.22 mm
matrix	512 *512
Slice thickness	5 mm
And field of view	350*350 mm

**Fig. 1:**
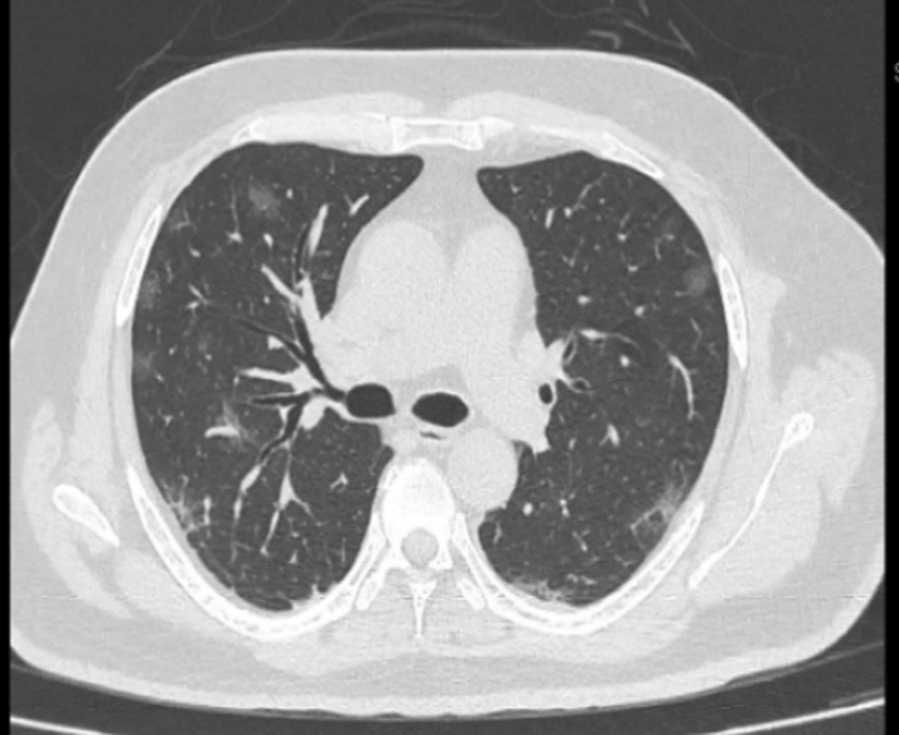
Axial CT image indicating mild disease severity (CTSS 5). Axial CT image shows GGO in bilateral upper and lower lobes mainly in subpleural and peripheral location with CTSS 5

**Fig. 2:**
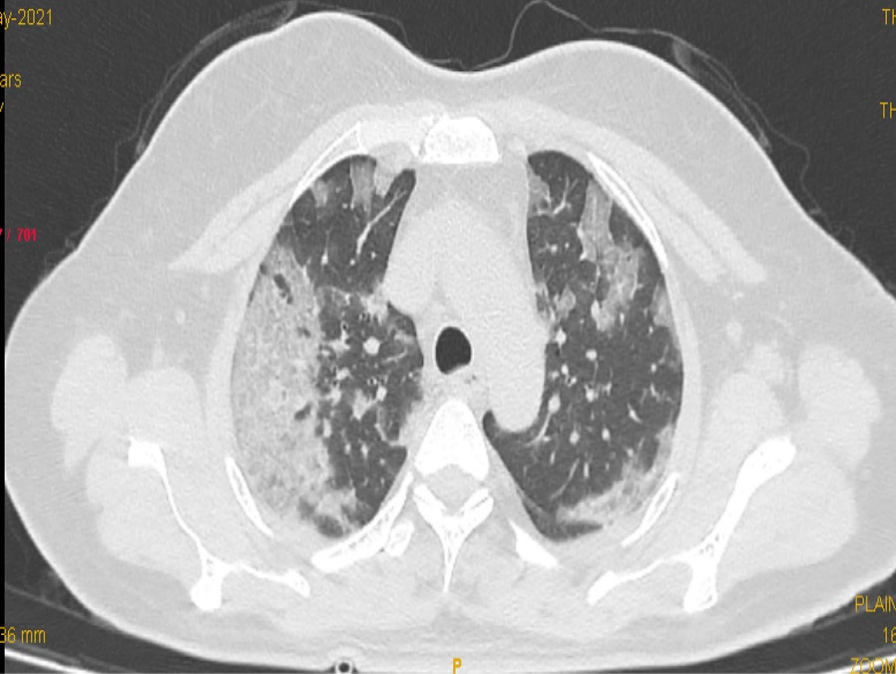
Axial CT image indicating moderate disease severity (CTSS 13). Axial CT image shows GGO with septal thickening and patchy consolidation in bilateral upper and lower lobes mainly in subpleural location with CTSS 13

**Fig. 3:**
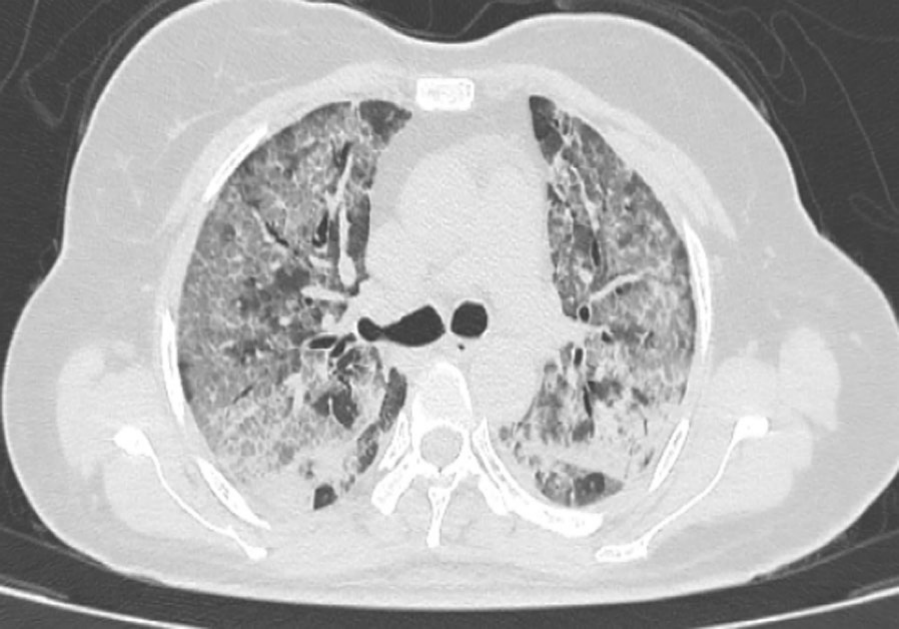
Axial CT image indicating severe disease severity (CTSS 20). Axial CT image shows extensive GGOs, crazy paving, and consolidation mainly in lower lobes bilaterally with CTSS 20

**Fig. 4:**
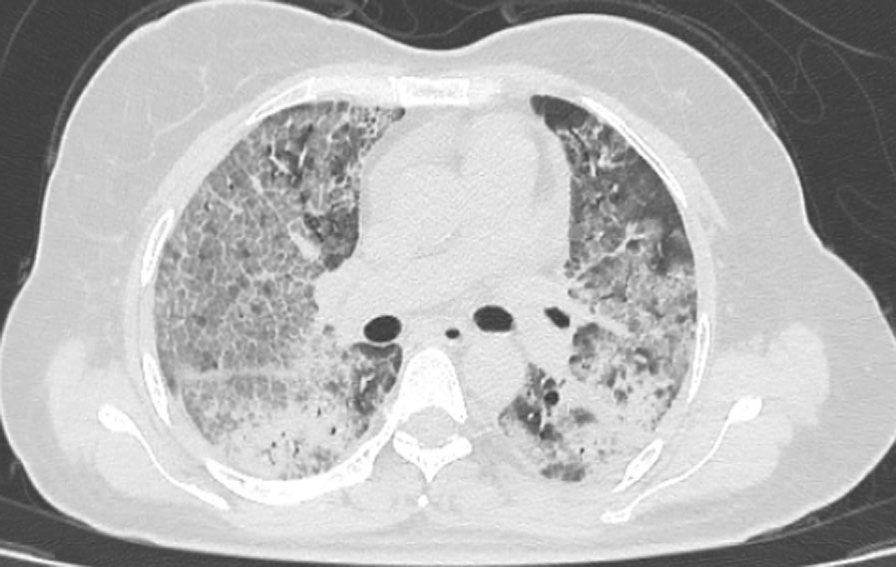
Axial CT image indicating severe disease severity with ARDS pattern (CTSS 25). Axial CT image shows diffuse extensive bilateral GGOs with crazy paving and multifocal consolidation bilaterally

The CT images were first evaluated for the presence of typical findings of COVID-19 pneumonia (subpleural unilateral or bilateral GGOs in the lower lobes with a peripheral or posterior distribution, which further develops into the crazy-paving pattern and subsequent consolidation) [8]. In all cases, a semiquantitative CT severity scoring proposed by Pan et al. [8] was calculated per each of the 5 lobes considering the extent of anatomic involvement, as follows [9]:

The 3 lung lobes on the right and 2 lobes on the left were individually assessed, and percentage involvement of the lobe was noted based on visual assessment. Visual severity scoring of CT chest was classified as Score-1 (< 5% area involved), Score-2 (5–25% area involved), Score-3 (25–50% area involved), Score-4 (50–75% area involved), Score-5 (> 75% area involved), making the total score 25. A CT severity score was assigned out of 25 based on the percentage area involved in each of the 5 lobes [7]. The total CT score is measured by the sum of the individual lobar scores and can range from 0 (no involvement) to 25 (maximum involvement), when all the five lobes show more than 75% involvement (see Figs. 1, 2, 3, 4).

Sum of individual lobar score indicates the overall severity of five lobes as detailed in Table 1.

**Table 1:** CT severity grading

Score	CT severity
< 8	Mild
9–15	Moderate
> 15	Severe

Frequency of CT severity scores was then compared with other clinical variables

### Statistical analysis

The data collected were formulated in a table and statistically analyzed.

Statistical tests were applied as follows.

- Quantitative variables—unpaired *t* test/Mann–Whitney test
- Qualitative variables correlated using Chi-square test/Fisher’s exact test

*p* value of < 0.05 was considered statistically significant.

## Results

The study population consisted of 150 laboratory-confirmed patients with COVID-19 disease. In this study, most of patients were in sixth and seventh decades with mean age of 51.12 years. Percentage distribution of patients according to age group was found as < 20 (0.7%), 21–40 (22.7%), 41–60 (36%), 61–80 (34.7%), > 80 (6%). Female patients (37.3%) were lesser than males (62.7). 55.2% patients had some or other underlying comorbid disease in sample population. These demographic characteristics are detailed in Table 2.

**Table 2:** Demographic and baseline characteristics of patients (*N* = 150)

Age of the patients	
20 years or less	1 (0.7%)
21–40 years	34 (22.7%)
41–60 years	54 (36.0%)
61–80 years	52 (34.7%)
Above 80 years	9 (6.0%)
Mean age (SD)	54.46 (16.71)
*Sex of the patients*	
Male	94 (62.7%)
Female	56 (37.3%)
*Type of comorbidity*	
CAD	8 (5.3%)
Diabetes mellitus	26 (17.3%)
COPD	15 (10.0%)
CKD	9 (6.0%)
CVD	5 (3.3%)
HTN	18 (12.0%)
Postpartum	2 (1.3%)

The most common comorbidity present in the study group was diabetes mellitus, which was present in 17.3% cases. 12% cases had hypertension, while 10% cases had chronic obstructive pulmonary disorder. 1.3% cases in the study were in their postpartum period.

### CT severity grading (Fig. 5)

**Fig. 5:**
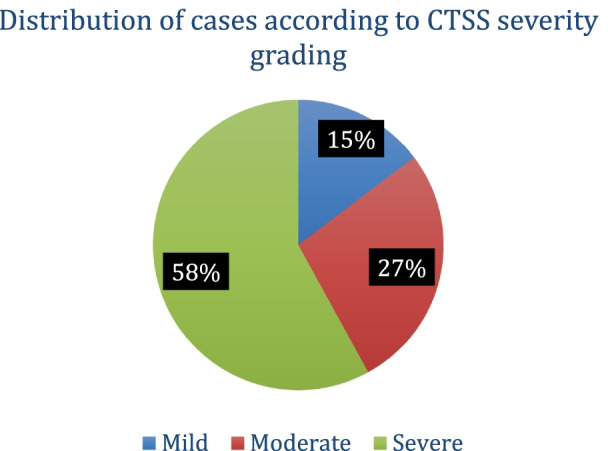
Distribution of cases according to CTSS severity grading

CT severity was graded as mild (grade 1) (< 8), moderate (grade 2) [10–16], and severe (grade 3) (> 15). 58% of sample population had grade 3 severity followed by moderate severity in 27.3% patients, and grade 1 severity was present in only 14.7% patients.

### Laboratory tests (Table 3, Fig. 6)

**Table 3:** Distribution of cases according to biochemical parameters (*N* = 150)

Parameters	Number of cases (%)	
	Normal	Increased
CRP	6 (4.0%)	118 (78.7%)
ESR	7 (4.7%)	103 (68.7%)
WBC	60 (40.0%)	73 (48.7%)
D-Dimer	28 (18.7%)	93 (62.0%)
S. Ferritin	34 (22.7%)	43 (28.7%)
Absolute lymphocyte count	78 (52.0%) (decreased)	59 (39.3%) (normal)

**Fig. 6:**
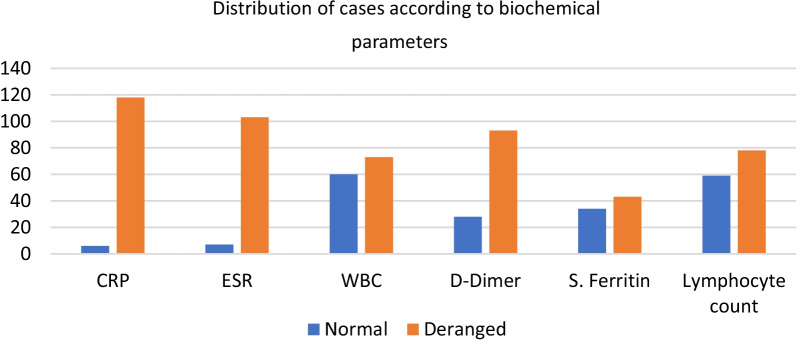
Distribution of cases according to biochemical parameters

Laboratory results showed raised CRP(CRP > 0.1 mg/dL) in 78.7% patients, raised ESR(ESR > 10 mm per hour) in 68.7%, lymphopenia (< 1–3 × 10^3^/µL) in 52% cases, increased D-dimer (> 0–200 ng/mL) in 62% patients, leucocytosis (WBC count > 4–10 × 10^3^/µL) in 48.7% patients, and increased serum ferritin (> 23.9–336 ng/mL) in 28.7% patients.

### Correlation between clinical parameters and CTSS (Table 4)

**Table 4:** Comparison of CT severity score according to demography and clinical parameters in all patients (*N* = 150)

Age group	No. of cases (%)			*p* value
	CTSS = mild	CTSS = moderate	CTSS = severe	
21 – 40 years	9 (42.85%)	11 (26.82%)	14 (16.09%)	0.049
41 – 60 years	4 (19.04%)	15 (36.58%)	35 (40.22%)	
61 – 80 years	8 (38.09%)	13 (31.7%)	31 (35.63%)	
Above 80 years	0 (0%)	2 (4.87%)	7 (8.04%)	
*Gender*				
Male	10 (45.45%)	27 (65.85%)	57 (65.51%)	0.195
Female	12 (54.54%)	14 (34.14%)	30 (34.48%)	
*Comorbidity*				
Present	10 (45.5%)	16 (39.0%)	33 (37.9%)	0.811
Absent	12 (54.5%)	25 (61.0%)	54 (62.1%)	

Mild disease was detected mainly in 21–40 year age group (42.8%) and least in > 80 years (0%). Moderate disease was detected mainly in 41–60 year age group (36.5%) and least in above 80 years (4.8%). Severe disease was detected mainly in 41-60 yr age group (40.2%) and least in above 80 years (8.04%). This study showed that severity of disease was significantly associated with increasing age of the patient. A higher proportion of cases with mild disease had a younger age, compared to severe disease, which was present among older age group.

Severity of disease was not significantly associated with sex of the patient. The distribution of cases with moderate and severe disease was comparable, with a higher proportion of male patients in both groups. Mild cases had a higher proportion of female patients, but this difference was not statistically significant.

The distribution of cases with comorbidity was comparable across all three grades of CT severity. Although mild cases (45.5%) had a higher proportion of patients with comorbidity, the difference was not statistically significant.

### CT severity score–laboratory test correlation (Table 5)

**Table 5:** Comparison of CT severity score according to biochemical parameters. (*N* = 150)

Biochemical markers	Mean (SD)			*p* value
	CTSS = mild	CTSS = moderate	CTSS = severe	
Lymphocyte count (× 10^3^/µL)	1.81 (1.57)	1.19 (0.88)	0.90 (0.95)	0.002
CRP (mg/dl)	5.35 (7.61)	5.24 (7.07)	11.71 (9.88)	0.045
ESR (mm 1^st^ hr)	49.35 (37.14)	42.91 (26.63)	60.42 (33.96)	0.143
Leukocytes (× 10^3^/µL)	10.76 (5.36)	12.48 (16.85)	16.16 (12.05)	0.173
D-dimer (ng/mL)	861.16 (1263.38)	1035.19 (1860.97)	2476.85 (5462.89)	0.002
S. Ferritin (ng/mL)	202.31 (237.41)	571.25 (478.15)	746.21 (486.81)	0.001

The above table shows the distribution of cases according to disease severity and biochemical parameter

Mean of lymphocyte count was 1.81 in mild group of patients, 1.19 in moderate group, and 0.90 in severe group. On comparison with disease severity as per CTSS, mean of lymphocyte count shows decreasing trend with increasing disease severity with significant statistical correlation (*p* = 0.002).

Mean of CRP was significantly higher in severe group (11.7) as compared to mild (5.3) and moderate (5.2) group. This finding was also found to have positive statistically significant correlation with CTSS (*p* = 0.045).

Mean of leucocyte count was 10.7 in mild group of patients, 12.4 in moderate group, and 16.16 in severe group. On comparison with disease severity as per CTSS, mean of leucocyte count shows increasing trend with increasing disease severity, however, with insignificant statistical correlation (*p* = 0.173).

Mean of ESR count was 49.3 in mild group of patients, 42.9 in moderate group, and 60.4 in severe group. This was found to be statistically insignificant on comparison with CTSS (*p* = 0.143).

Mean of D-dimer values was 861.16 in mild group of patients, 1035.19 in moderate group, and 2476.85 in severe group. On comparison with disease severity as per CTSS, mean of D-dimer shows increasing trend with increasing disease severity, with positive statistical correlation (*p* = 0.002).

Mean of serum ferritin was 202.31 in mild group of patients, 571.25 in moderate group, and 746.21 in severe group. On comparison with disease severity as per CTSS, mean of serum ferritin shows increasing trend with increasing disease severity, with positive statistical correlation (*p* = 0.001).

### CT severity–patient survival correlation (Table 6)

**Table 6:** Distribution of cases according to CT severity score and survival (*N* = 150)

CTSS	Number of cases (%)		*p* value
	Patients died (*N* = 75)	Patients survived (*N* = 75)	
Mild	1 (1.3%)	21 (28.0%)	0.001
Moderate	12 (16.0%)	29 (38.7%)	
Severe	62 (82.7%)	25 (33.3%)	
Mean (SD)	20.39 (4.51)	12.89 (7.12)	0.001

Among the patients who died (75, 50%) of COVID-19 disease, 82% had severe disease, and 16% had moderate grade disease, while only 1.3% has mild grade disease. This suggested that a significantly higher proportion of cases who did not survive had a severe grade of disease on CT scan. In contrast, the proportion of cases who survived was comparable among all grades of severity on CT scan. This difference between the two groups was statistically significant (*p* = 0.001). The mean CT severity score among patients who died was significantly higher than patients who survived.

## Discussion

Coronavirus disease 2019 (COVID-19) is a highly infectious viral respiratory disease that has recently emerged from China and has become a pandemic [3].

The WHO recommends chest imaging in suspected COVID-19 patients when RT-PCR test is not available or in patients with negative test results [17, 18].

In the present study, an attempt was made to outline distribution of age, gender, clinical, and laboratory features at presentation, comorbidity of patients, severity of patients on the basis of CT imaging, and their correlation with clinical and laboratory parameters of patients to put diagnostic, therapeutic, and prognostic tools for COVID-19 disease [7].

Majority of cases were aged between 41 and 60 years, followed by 61–80 years. Only one case was aged 18 years, who was also the youngest participant in the study. The eldest participant was aged 86 years. Mean age of the study group was 54.46 (± 16.71) years.

Among previous studies, Bhandari S et al. [7] reported that mean age of the patients was 50.40 years, which is very similar to our study.

This study showed statistically significant correlation between severity of disease and increasing age of the patient.

A higher proportion of cases with mild disease had a younger age, compared to severe disease, which was present among older age group. This can be attributed to different factors like patients comorbidities, preparation of healthcare system, and stage of pandemic [19].

In present study, female patients were lesser than males, where almost two-third of cases were male patients, which is similar to previous study done by Bhandari S. [7]. This may be due to gender bias or due to the reduced susceptibility of females to viral infections which might be attributed to the protection from X chromosome and sex hormones, which play an important role in innate and adaptive immunity [2, 17]. There was no significant gender differentiation of CT chest findings and laboratory parameters. Also, severity of disease was not significantly associated with sex of the patient. The distribution of cases with moderate and severe disease was comparable, with a higher proportion of male patients in both groups. Mild cases had a higher proportion of female patients, but this difference was not statistically significant.

Among all patients, 55.2% patients had some or other underlying comorbid disease like diabetes mellitus, hypertension, COPD, and others. Although in this study, there was a higher proportion of cases with comorbidity in mild grade of disease, the difference was not statistically significant. This is similar to previous study done by Saeed G [20].

In this study, patient survival was significantly decreased among patients with severe CT findings. In contrast, the proportion of cases who survived was comparable among all grades of severity on CT scan. This difference between the two groups was statistically significant. The mean CT severity score among patients who died was significantly higher than patients who survived.

### CT severity score and CT severity grading

According to the degree of lung involvement evaluated by CT severity score (CTSS) of all the lobes of both lungs, the patients were classified on the basis of severity of CTSS score as mild (grade1), moderate (grade 2), and severe (grade 3). In our study population, more than half of the cases had a severe grading on CT scan. More than a quarter of the cases were labeled as moderately severe on CT score, while 22 cases had mild severity. This observation could be due to sample being taken from tertiary care hospital which caters comparatively sick patients.

### Laboratory parameters

Many previous studies have suggested alterations of laboratory parameters in COVID-19 patients with greater frequency such as lymphocyte count, CRP, D-dimer, and serum ferritin [21]. In our study, more than three-fourth of the cases has a raised CRP level (CRP > 0.1 mg/dL), while more than two-thirds of the cases had a raised ESR level (ESR > 30). In contrast, almost comparable proportion of cases had raised and normal absolute leukocyte count and serum ferritin. D-dimer levels were raised in majority (62.0%) of the cases. Lymphocyte count was decreased in more than half (52%) of the cases.

This study also correlated laboratory parameters with CT severity score. This associates laboratory parameters with CT and RT-PCR tests to play an important role in the diagnosis and management of highly suspicious patients of SARS-CoV-2 infection [21].

Severe disease was associated with a significantly lower lymphocyte count compared to mild disease. In contrast, CRP, D-dimer, and serum ferritin were significantly higher in severe cases compared to mild cases. Strong correlation between lymphopenia and disease severity can be related to the inflammatory cytokine storm in COVID-19 patients [20].

This study showed statistically significant correlation between raised CRP levels and increasing CT severity. Previous studies have also suggested that CRP can be used as a predictive marker for likelihood of disease progression and can guide clinicians in early treatment at early disease stage. Similarly, serum ferritin acts as a vital mediator of immune dysregulation as shown in this study where serum ferritin level was closely correlated with severity of disease [20].

Likewise, higher D-dimer levels were seen in severe disease and can be used as a prognostic indicator.

Mortality rate of COVID-19 patients in this study was associated with higher CT severity grade. The mean CT severity score among patients who died was significantly higher than patients who survived.

### Limitations of the study

First, this is a modest-sized study of patients admitted to the hospital, and a larger cohort is required to obtain a definitive answer.

Second, the quantitative and semiquantitative methods used measuring the pulmonary involvement and severity may have certain subjectivity.

Third, the susceptibility of COVID-19 was considered to be low among infants, children, and adolescents, so we did not retrospectively study these groups.

## Conclusions

In conclusion, chest CT severity score of patients with COVID-19 is positively correlated with inflammatory laboratory markers and hence can work as an indicator of disease severity and outcome. CT chest imaging can play a vital role in the management plan of COVID-19 pneumonia and should be used for comprehensive evaluation, combined with the results of nucleic acid tests and the epidemiological data.

## Data Availability

The datasets used and/or analyzed during the current study are available from the corresponding author on reasonable request.

## Abbreviations

CT: Computed tomography
COVID-19: Coronavirus disease 2019
SARS-CoV-2: Severe acute respiratory syndrome coronavirus 2
CRP: C reactive protein
nCoV: Novel coronavirus
RT-PCR: Reverse transcription polymerase chain reaction
CTSS: Computed tomography severity score
ESR: Erythrocyte sedimentation rate
S. ferritin: Serum ferritin
